# Effect of Vitamin D Deficiency on Liver Cancer Risk: A Systematic Review and Meta-Analysis

**DOI:** 10.31557/APJCP.2021.22.4.991

**Published:** 2021-04

**Authors:** Zhenghui Yi, Linjie Wang, Xiangqun Tu

**Affiliations:** 1 *Department of General Surgery, Civil Aviation General Hospital, No.1 Gaojing, Chaoyang Street, Beijing, China. *; 2 *Key Laboratory of Endocrinology of National Health Commission, Department of Endocrinology, Peking Union Medical College Hospital, Chinese Academy of Medical Science and Peking Union Medical College, Beijing, China. *

**Keywords:** 25-hydroxyvitamin D, liver cancer, meta-analysisRessundit, omnimust inctore mpellore expel eos

## Abstract

Epidemiological studies have showed that vitamin D deficiency can increase the risk of liver cancers. Hence, we conducted a meta-analysis to explore the relationship between 25-hydroxyvitamin D [25(OH)D] levels and liver cancer risk.

**Methods:**

Cochrane Library, Medline, Web of Science, and Embase were searched up to Mar. 2020, and the references of those studies were also searched by hand. A meta-analysis of 11 studies was performed which met the inclusion criteria. Six case–control studies and five cohort studies were included.

**Results:**

A total of 11 studies (6 case–control and 5 cohort studies) with 12,895 incident cases were included in the meta-analysis. The meta-analysis showed that liver cancer risk was significantly increased for vitamin D deficiency, and the pooled RR and its 95% CIs was 2.16 (1.2, 3.88; P = 0.01). In comparative analyses between 25(OH)D levels in patients with hepatocellular carcinoma(HCC) and those in the control group individuals, the summary RR of liver cancer was -1.11 (95% CI=-1.96 to -0.25). The subgroup analysis of the different geographical region of the population showed that the risk of liver cancer in Asian subgroup, European subgroup and Egyptian subgroup increased for vitamin D deficiency (RR=1.34,95% CI 0.72 to 2.48, P<0.00001; RR=2.53,95% CI 1.62 to 3.93,P<0.0001;RR=29.5,95% CI 4.14 to 209.93, P=0.88).

**Conclusion:**

The results of this meta-analysis indicate that vitamin D deficiency is associated with increased risk of liver cancer. The 25(OH)D3 levels are lower in HCC patients than those in health controls. Maintenance of sufficient serum vitamin D levels would be beneficial for prevention of liver cancer.

## Introduction

Primary liver cancer is the second leading cause of cancer-related death worldwide (Freddie et al., 2018). Incidence of liver cancer remains highest in Asia, approximately reaching 78% of all, specifically in the East and South-East Asia (Jessica et al., 2019). Risk factors for liver cancer include chronic infection with hepatitis B or C virus (HBV or HCV), excess alcohol consumption, aflatoxin exposure, diabetes, obesity, and smoking (Hana et al., 2020). Many clinical reports have demonstrated that vitamin D deficiency is very common in patients with liver cancer(Ashish et al., 2018). Experimental studies also confirmed that vitamin D is related to poor tumor response and the prognoses of patients with liver cancer(Wu et al., 2019).

Vitamin D is an essential nutrient that regulates numerous cellular pathways. Many cell signaling pathways activated by vitamin D are related with cancer development, progression and prognosis (Xu et al., 2020; Wang et al., 2018). Vitamin D deficiency increases the risk of fracture risk, cardiovascular disease, hypertension, diabetes mellitus, and cancers (Fatemeh et al., 2019; Hala et al., 2019; Larsen et al., 2012). Indeed, the biological activity of vitamin D is regulated by a complicated feedback regulation system and depends on receptors of vitamin D(VDR) and other transport proteins. Vitamin D and VDR are not only involved in the immune system (Watkins et al., 2015), but also playing important roles in the modulation of tumor growth and metastasis (Johnson et al., 2015; Williams et al., 2016). Vitamin D deficiency and insufficiency is a global phenomenon, and it has been estimated that approximately 60% of adults worldwide are vitamin D deficient and insufficient (Daly et al., 2012). It is known that Vitamin D deficiency is particularly prevalent in South Asian, including severe deficiency (<12.5 nmol/l) up to 27–60% of individuals (Darling, 2020). In India, vitamin D deficiency prevails in epidemic proportions, with a prevalence of 70%-100% in the general population (Ritu and Ajay, 2014). The major causes for the vitamin D deficiency pandemic are the lack of sun exposure and inadequate intake of natural dietary sources (Chang and Lee, 2019). If the status of vitamin D deficiency is not corrected, it may promote liver cancer growth through β-catenin activation and toll-like receptor 7 signaling pathways (Chen et al., 2016). In the past decade, many studies have demonstrated that vitamin D deficiency is significantly associated with incident liver cancer (Wang et al., 2013; Targher and Byrne, 2014), however, the findings remain controversial. Therefore, based on prospective cohort and case-control studies, we conducted a meta-analysis to systematically assess the relationship of vitamin D levels with the risk of liver cancer.

## Materials and Methods

*Search strategy*

All relevant studies were systematically searched in PubMed, Embase, Web of Science and Cochrane Library before March 2020. No restrictions for time of publish were imposed, except for human subjects and English language. Moreover, the reference lists from previous systematic reviews and published meta-analyses were reviewed. Abstracts, meeting proceedings, gray literature, unpublished results, and personal communications were not included. 

*Study selection*

Studies were eligible for inclusion if they 1) applied a case–control, cohort study, or clinical trial design and were published as original studies; 2) assessed at least one of the following exposures: vitamin D or 25(OH) D3; 3) use liver cancer or hepatocellular carcinoma as an outcome; and 4) provided at least two groups of relative risks (RRs) or odds ratios (ORs) and 95% confidence intervals (95% CIs) across different categories or could estimate these data by number of cancer cases and population at risk (or controls). To avoid overlap between study populations, we included studies with the largest sample size when studies on the same topic were performed among the same populations. 

*Quality assessment*

The Newcastle–Ottawa Scale (NOS) was used to assess the quality of the included original studies with a case–control or cohort study design (Stang, 2010). The content of the study was evaluated for four major aspects: selection, comparability, exposure, and results, and thereafter, categorized into high, medium, and low quality. A study with a score > 6 was considered to be well quality. 

*Data extraction*

The titles and abstracts of the initially retrieved articles were independently screened by two investigators (YZH and WLJ). Disagreements between the two authors will be solved through

discussion with the co-author (TXQ) to reach a consensus. The following data from each eligible study were extracted: the first author, publication year, country of the study, study name, study design, characteristics of the study population, follow-up period, sample size, race, age, number of cases, blood vitamin D levels, testing method, maximally adjusted ORs or RRs with 95% CIs, and adjusted covariates.

*Statistical analysis*

Statistical analysis was performed with Review Manager 5.3 (The Nordic Cochrane Centre, Copenhagen, Denmark) from The Cochrane Collaboration. Continuous variables were analyzed using mean differences (MD) and 95 % confidence intervals (CIs). A P value <0.05 was considered statistically significant. Heterogeneity of the included studies was tested by using Higgins I^2^[I^2^= (Q− df)/Q × 100], with a significance threshold of I^2^>50 % (Higgins and Thompson, 2002). A random effects model was used if the I^2^ statistic was significant. Otherwise, a random effects model was employed. Potential publication bias was assessed by using Begg’s test through using the Stata 16 software. 

## Results

*Selection of studies*

**Figure 1:**
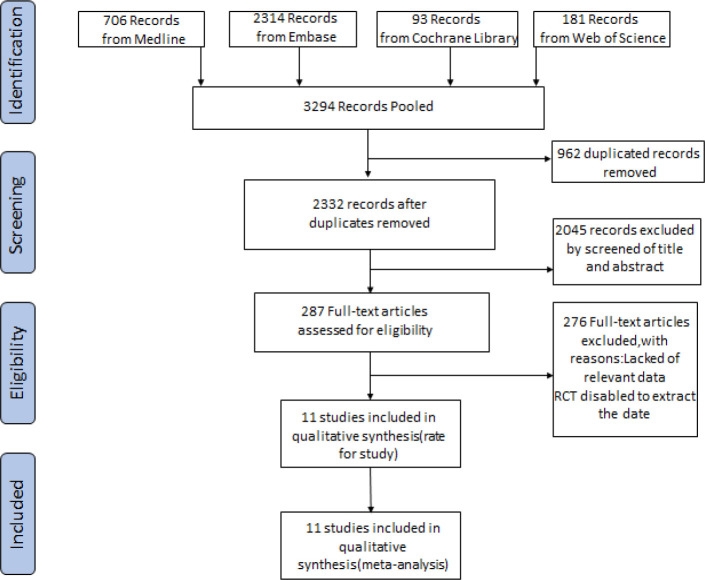
flow Diagram of Literature Search

A preferred reporting items for meta- analyses flow diagram of literature search is shown in Figure 1. The initial literature identified 3,294 titles from PubMed (n=706), EMBASE (n=2314), Science direct (n=181) and the Cochrane Library (n=93). We excluded 2,045 more articles after carefully reading the abstracts and another 1,236 because they were duplicates, abstracts of meetings, reviews, case reports, or did not meet the selection criteria. Ultimately, 11 studies were selected for meta-analysis.

*Study characteristics*

The eleven studies included five cohort studies and six case-control studies. The five cohort studies (Wong et al., 2015; Sanjeev et al., 2018; Finkelmeier et al., 2014; Buonomo et al., 2019; Fang et al., 2019) consisting of 9,347 incident cases, and 6 case-control studies (Wang et al., 2013; Nghiem et al., 2016; Hammad et al., 2013; Veronika et al., 2014; Schaalan et al., 2012; Gabriel et al., 2018) consisting 1723 cases and 1825 controls were included. Among these, 4 studies were conducted in the Europe (Finkelmeier et al., 2014; Buonomo et al., 2019; Veronika et al., 2014; Gabriel et al., 2018), 2 in the Egypt (Hammad et al., 2013; Schaalan et al., 2012), 5 in Asia (Wang et al., 2013; Wong et al., 2015; Sanjeev et al., 2018; Fang et al., 2019; Nghiem et al., 2016). The included studies, basic characteristics of enrolled patients, and surgical techniques are presented in Table 1.

*Overall analyses*

*Vitamin D deficiency and liver cancer risk*

**Figure 2:**
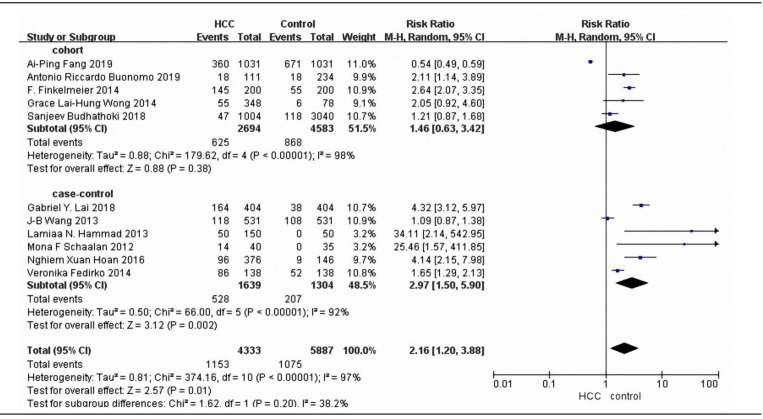
Meta-Analysis of Association between Blood Circulating Vitamin D and the Risk of Liver Cancer. The adjusted RRs of liver cancer between vitamin D deficiency and vitamin D sufficiency from each study were included. Abbreviation:RR, relative risk; 95%CI, 95% confidence intervals

In comparative analyses between vitamin D deficiency and vitamin D sufficiency, significantly increased liver cancer risk was observed for vitamin D deficiency, and the pooled RR and its 95% CIs was 2.16 (1.2–3.88; P=0.01) with moderate heterogeneity (I^2^=97%) (Figure 2). Six case-control studies and five cohort studies were eligible for the analysis of the association of vitamin D deficiency and liver cancer risk. As shown in Figure 2, the random-effects model was used and showed that vitamin D deficiency had no significant effect on occurrence of liver cancer in cohort subgroup, and the pooled RR were 1.46 (95% CI =0.63–3.42,I2=98%,P=0.38) for cohort studies. In case-control subgroup, there was a significant effect on occurrence of liver cancer, and the pooled RR were 2.97(95% CI=1.5–5.9, I^2^=92%, P=0.002) for case-control studies. This result suggests that vitamin D deficiency could increase the risk of liver cancer.

*Serum 25(OH)D levels and liver cancer risk*

**Figure 3:**
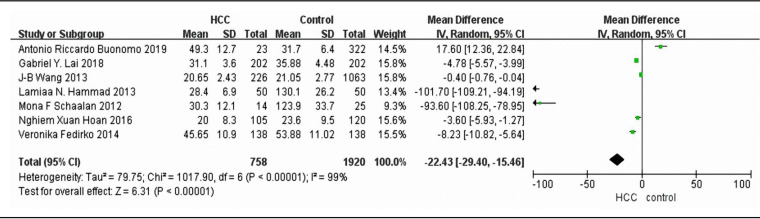
Meta-Analysis of Association between Blood Circulating vitamin D and the Risk of Liver Cancer

The summary RR of liver cancer for the HCC patients versus control individuals of blood vitamin D levels was -1.11 (95% CI=-1.96 to -0.25), with an evidence of heterogeneity I^2^=98%, P<0.00001 , indicating that serum 25(OH)D level in HCC patients was 1.11nmol/L lower than that in control group (Figure 3). Additionally, we did not analyze case–control and cohort studies separately, because only one cohort study demonstrated the mean blood circulating vitamin D level.

*Subgroup analysis*

**Figure 4:**
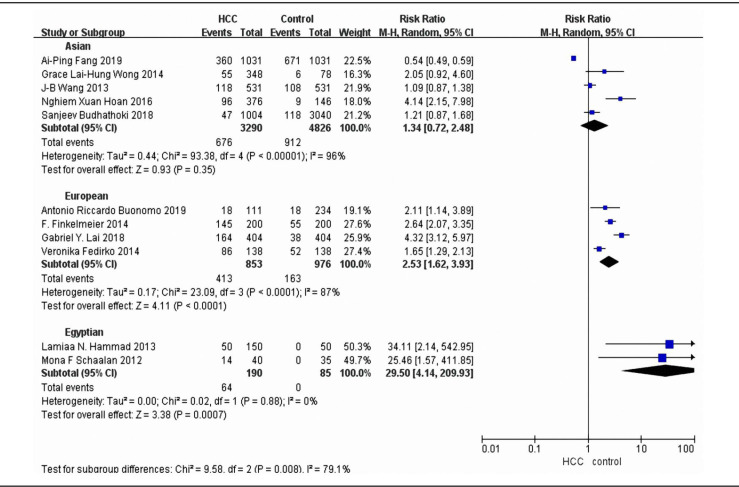
Subgroup Analysis of the Different Geographical Region in the Risk of Liver Cancer

We stratified subgroup analysis based on geographical region of the population (Figure 4). The pooled RRs were 1.34 (95% CI 0.72 to 2.48;P<0.00001) for eastern Asia, 2.53 (95% CI 1.62 to 3.93;P<0.0001) for Europe and 29.5 (95% CI 4.14 to 209.93; P=0.88) for Egypt. The subgroup meta-analyses are shown in Figure 4.

*Publication Bias*

**Figure 5:**
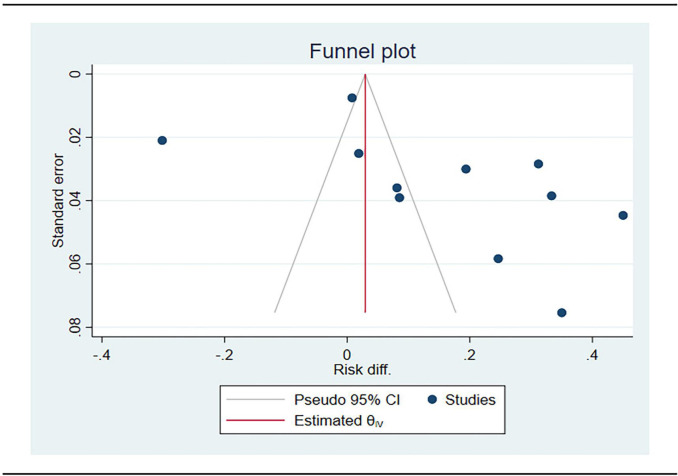
Begg's Funnel Plot

The funnel plot and Begg’s statistical test indicated no evidence of publication bias in the studies included in the meta-analysis (p=0.16) (Figure 5.) . 

**Table 1:** Basic Characteristics of Included Studies in the Meta-Analysis of Vitamin D and Liver Cancer

Year and first author of study	Country	Type of study	No. of Cases /Controls	Mean Age (years)	Sex	Blood sample type	Vitamin D testing method	Follow-up period (year)
2014, Grace Lai-Hung Wong	China	Cohort	348/78	41±13	All	serum	electrochemiluminescence binding assay	13±4
2013, J-B Wang	China	Case-control	508/1063	55	All	serum	enzyme immunoassay	1991-2007
2018, Gabriel Y. Lai	Finland	Case-control	427/427	NA	Men	serum	Direct competitive chemiluminescence immunoassay	15
2016, Nghiem Xuan Hoan	Vietnam	Case-control	400/122	46	All	serum	ELISA kit	NA
2013, Lamiaa N. Hammad	Egypt	Case-control	200/50	NA	All	serum	ELISA	NA
2018, Sanjeev Budhathoki	Japan	Cohort	3301/4044	NA	All	Plasma	chemiluminescent enzyme immunoassay	1990-2009
2014, Veronika Fedirko	Europe	Case-control	138/138	59.9	All	serum	liquid chromatography/tandem mass spectrometry	1992-2010
2019, Ai-Ping Fang	China	Cohort	360/671	53	All	serum	electrochemiluminescence immunoassay	2
2014, F. Finkelmeier	German	Cohort	145/55	63	All	serum	radioimmunoassay	1
2019, Antonio Riccardo Buonomo	Italy	Cohort	111/234	68	All	serum	NA	1
2012, Mona F Schaalan	Egypt	Case-control	50/25	42.5	All	serum	solid phase radioimmunoassay	7

All*, all participants, including men and women; NA, not available.

## Discussion

Vitamin D deficiency is known to be associated with the increased risk of many human cancers (Daniel, 2016; Wang et al., 2017; Feldman et al., 2014), and extensive epidemiological studies supported vitamin D–cancer hypothesis (Feldman et al., 2014; Grant, 2017). Hepatocellular carcinoma is the second most commonly diagnosed cancer and the leading cause of cancer death in men (Jemal, 2012).It’s life expectancy after diagnosis rarely exceeded 3 years in developed countries (Fitzmaurice et al., 2015). Several population-based studies unveiled an inverse correlation between serum 25(OH)D levels and high risk of liver cancer (Gabriel et al., 2018). Moreover, extensive animal and cell culture studies support the anti-tumorigenic effects of vitamin D (Ravid et al., 2019; Wang et al., 2018). As such, it seems that deficiency of vitamin D can contribute to the development and progression of liver cancers.Nevertheless, the evidence of the association of vitamin D with liver cancer risk remains controversial. Fang et al., (2019) shown that 25(OH)D levels were associated with risk of liver cancer in the population-based HCC cohort in China. Therefore, our meta-analysis aimed to explore the correct relationship between vitamin D and liver cancer risk. 

In this meta-analysis, we obtained summary evidence that vitamin D deficiency will increase the risk of liver cancer, and the pooled relative risk is 2.16. To further analyze the risk of liver cancer in two different research type, we calculated the RRs of cohort subgroup and case-control subgroup respectively. There are five cohort studies that combined in this project for meta-analysis, and the pooled relative risk is 1.46. Six case-control studies were included for subgroup meta-analysis, and the pooled relative risk is 2.97. In addition, our meta-analysis found 1.81 increased risk of liver cancer for the lowest versus highest categories of blood circulating vitamin D levels (RR=-22.43, 95% CI -29.4to -15.46), that indicated higher blood circulating vitamin D level has a significant inverse association with risk of liver cancer. This result was consistent with Guo’s meta-analysis (Guo et al., 2020). Guo et al., (2020) analyzed six prospective studies and reached the conclusion that a higher circulating vitamin D was associated with lower risk of liver cancer. Our meta-analysis results indicated that the negative correlation between vitamin D level and the risk of liver cancer is similar to that of European and Asian population studies (Sanjeev et al., 2018;Veronika et al., 2014).

When we stratified studies by geographical location, the result showed a statistically difference between Asian, European, and Egyptian. Because the presence of poor heterogeneity and the diversity of the studies in the overall analysis (I^2^=97%, Pheterogeneity<0.00001), it may not reflect the true underlying effect. The poor heterogeneity could be caused by the presence of season of blood collection, different races, enrolled individuals with viral hepatitis and the method for measuring blood vitamin D levels. In addition, few studies about this topic were eligible for our current meta-analysis. 

Vitamin D is an inactive steroid derivative that needs to be metabolized to bio-active products in the liver (Göring, 2018). First, vitamin D is metabolized by vitamin D 25-hydroxylase to 25(OH)D, which is the major circulating form of vitamin D in serum (Roizen et al., 2019; Jorde et al., 2018). The second step of activation occurs in the proximal tubular of kidney. To date, there are numerous methods to measure the serum 25(OH)D, however the serum 25(OH)D level can be affected by liver function, viral hepatitis and sunlight exposure(Gao et al., 2017). That may also affect our heterogeneity of meta-analysis.

There are some limitations in the present study. First, although we systematically searched four authoritative databases, there were still some missed studies that could have affected the analysis for unavoidable reasons. Second, We did not analyze the case–control and cohort studies separately. Third, since most of the included studies were performed without American populations, it is not sure whether our findings could be generalized to global populations. 

In conclusion, our meta-analytical comparison supports the hypothesis that vitamin D deficiency status is related to a higher risk of liver cancer, and thus maintenance of sufficient serum vitamin D levels could be beneficial for prevention of liver cancer. Further multinational, population-based study will be need to resolve the issue of heterogeneity and evaluate the association of blood circulating vitamin D levels and the risk of liver cancer.

## Author Contribution Statement

All authors read, critically reviewed and approved the final manuscript. YZH and WLJ conducted the database searches, screened titles, abstracts and full-texts for eligibility, performed study quality assessments. YZH planed and designed the research; WLJ and TXQ provided methodological support/advice; YZH tested the feasibility of the study; YZH and WLJ extract data; YZH performed the statistical analysis; YZH wrote the manuscript. 
